# The temporal relationship between poor lung function and the risk of diabetes

**DOI:** 10.1186/s12890-016-0227-z

**Published:** 2016-05-10

**Authors:** Suneela Zaigham, Peter M. Nilsson, Per Wollmer, Gunnar Engström

**Affiliations:** Department of Clinical Sciences Malmö, Lund University, CRC 60:13, Jan Waldenströms gata 35, S-20502 Malmö, Sweden; Department of Translational Medicine, Lund University, Malmö, Sweden

**Keywords:** Diabetes, Incidence, Lung function

## Abstract

**Background:**

The association between impaired lung function and diabetes risk has been established in the past, however the temporal and causal relationships between the two remain unclear. We assessed the relationship between baseline FEV_1_ and FVC and risk of incident diabetes at different time intervals for participants in the Malmö Preventive Project cohort.

**Methods:**

Baseline lung function was assessed in 20,295 men and 7416 women during 1974–1992; mean age 43.4 ± 6.6 and 47.6 ± 7.8, respectively. Sex-specific quartiles of FEV_1_%predicted and FVC%predicted were created (Q4 = highest; reference). Follow-up time was divided into 10-year time intervals from baseline examination. Cox proportional hazards regression was used to assess the incidence of diabetes according to quartiles of FEV_1_ and FVC%predicted, after adjustments for baseline glucose and potential confounding factors.

**Results:**

Over 37-years’ follow-up there were 3753 and 993 incident diabetes events in men and women, respectively. When comparing FEV_1_%predicted in men (Q1 vs. Q4), the HR for diabetes was 1.64 (1.21–2.22) for events <10 years after baseline, 1.52 (1.27–1.81) for events 10–20 years after baseline, 1.39 (1.22–1.59) for events 20–30 years after baseline, and 1.46 (1.08–1.97) for events occurring >30 years after baseline. A broadly similar pattern was seen for FVC%predicted and for women.

**Conclusions:**

Low FEV_1_ precedes and significantly predicts future diabetes. This risk is still significant many years after the baseline FEV_1_ measurement in middle-aged men. These results suggest that there is a relationship between impaired lung function and diabetes risk beyond the effects of hyperglycemia on lung function.

**Electronic supplementary material:**

The online version of this article (doi:10.1186/s12890-016-0227-z) contains supplementary material, which is available to authorized users.

## Background

Poor lung function has been long found to have a relationship with important health outcomes beyond the effect of smoking [1]. One of these important relationships is the complex interaction between diabetes and lung function. Although cross-sectional studies have found that patients with diabetes tend to have poorer lung function than non-diabetics [2–6], the temporality of a causal relationship has remained controversial in longitudinal studies. Some longitudinal studies have explored the relationship between diabetes and lung function decline later in life and found an association [3, 7], whereas others have not [5, 8]. The proposed mechanisms have included microangiopathy of lung vasculature, chronic inflammation, autonomic neuropathy involving the lung, and loss of elastic recoil due to glycosylation of lung parenchyma, i.e. adverse effects of high glucose levels [9]. However, more recently poor lung function has been thought to be a potential novel risk marker for future diabetes and there have been a number of longitudinal studies that have explored poor lung function at baseline and the future risk of diabetes [10–18]. The relationship between impaired lung function and diabetes is important to explore as further understanding of this relationship can help inform strategies to impact the burden of significant conditions related to both poor lung function and diabetes, such as cardiovascular disease (CVD). Stratifying a long-term prospective analysis into different follow-up periods can help further understand the temporal relationship between diabetes and lung function.

We have previously used the Malmö Preventive Project (MPP) cohort to report that subjects with a moderately reduced forced vital capacity (FVC) at baseline run an increased risk of developing insulin resistance and diabetes after 14-years of follow-up [11]. The aim of the present longitudinal cohort study is to add clarity to the temporal relationship between poor lung function and diabetes in a prospective study from the general population. We aim to do this by firstly establishing if poor lung function (as defined by low %predicted forced expiratory volume in one second (FEV_1_) or FVC) is a predictor of future diabetes and secondly if so, how long time-wise from the baseline lung function measurement this risk can be observed. As systemic inflammation is often thought to be a common factor that links both poor lung function and diabetes, we also aim to analyse if the relationship is affected by various inflammatory factors.

## Methods

### Study population

The study population for the present study is derived from the MPP. A total of 33,346 subjects (22,444 men and 10,902 women) participated in screening activities between 1974 and 1992 (attendance rate over 70 %), with an aim to screen a large population of middle-aged individuals and offer preventive treatment to any high risk individuals identified during screening. Complete birth cohorts, born between 1921 and 1949, were invited for health screening which included a physical examination, a panel of laboratory tests, spirometry and a self-administered questionnaire.

The Health Service Authority of Malmö approved the screening program, and linkage with the national cause of death and patient registers was approved by the Regional ethics committee at Lund University. Men were mostly screened during 1974–82 and women were mostly screened during 1982–92. From the total study population those with a history of diabetes at baseline (prevalent diabetes) were excluded (*n* = 1173). Spirometry was performed in birth cohorts during most but not all screening time periods (94 % of men and 71 % of women underwent spirometry) however individuals were not selected based on symptoms or disease. Those with missing FVC or FEV_1_ were then excluded (*n* = 4149) from the study. Individuals with missing information on baseline glucose (*n* = 120), height (*n* = 1), BMI (*n* = 2), erythrocyte sedimentation rate (ESR) (*n* = 58), smoking status (*n* = 5), cholesterol (*n* = 38), or information on family history of diabetes (*n* = 31) were also excluded. We also excluded individuals with an ESR ≥50 mm/h as this may indicate any specific inflammatory lung pathology (*n* = 58). The final study population thus consisted of 27,711 subjects (20,295 men and 7416 women).

**Fig. 1:**
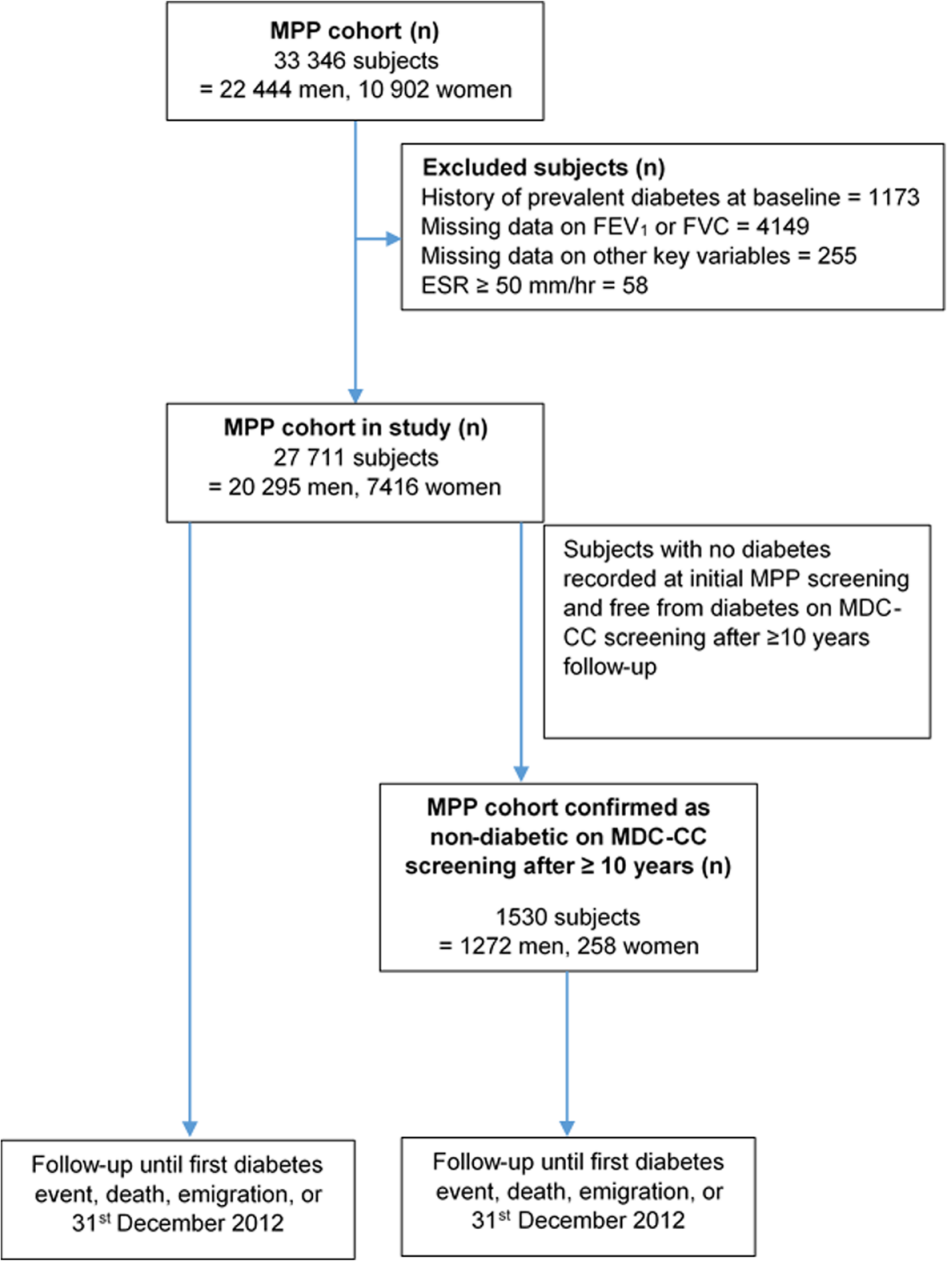
Illustrates the flow of subjects from MPP cohort and MDC-CC cohort

Since diabetes develops gradually and can go undetected for a long time, we performed a subgroup analysis of individuals who were confirmed to be non-diabetic at least 10 years after the baseline screening. Between 1992 and 1994, the cardiovascular cohort of the Malmö Diet and Cancer study (MDC-CC) was performed, which included measurements of fasting blood glucose [19]. Of the 27,711 subjects in the present study, 2539 men and women participated also in the MDC-CC. Of them, 1530 individuals were examined at least 10-years after the initial screening in the MPP cohort and were non-diabetic when they were re-examined in the MDC study (i.e. fasting blood glucose <6.1 mmol/L, no self-reported diabetes or anti-diabetic medication). Figure 1 illustrates the flow of subjects through the study.

### Baseline examinations

FEV_1_ and FVC were measured using a Spirotron apparatus (Drägerwerk AG, Lübeck, Germany) carried out by trained nursing staff. One acceptable manoeuvre was required. FEV_1_ and FVC were standardised for age and height using published equations derived from linear regression of never-smokers in the present cohort [20–22].

The equations used for computing predicted values were as follows:

$$ \begin{array}{l}\mathrm{Men}:\hfill \\ {}\mathrm{Predicted}\kern0.5em {\mathrm{FEV}}_1\left(\mathrm{L}\right):4.422\times \mathrm{height}\kern0.3em \left(\mathrm{m}\right)\kern0.3em -\kern0.3em 0.0381\times \mathrm{age}\kern0.3em \left(\mathrm{years}\right)\kern0.3em -\kern0.3em 2.483\hfill \\ {}\mathrm{Predicted}\kern0.5em \mathrm{F}\mathrm{V}\mathrm{C}\left(\mathrm{L}\right):6.58\times \mathrm{height}\kern0.1em -\kern0.1em 0.033\times \mathrm{age}\kern0.1em -\kern0.1em 5.54\hfill \end{array} $$

$$ \begin{array}{l}\mathrm{Women}:\hfill \\ {}\mathrm{Predicted}\kern0.5em {\mathrm{FEV}}_1\left(\mathrm{L}\right):3.615\times \mathrm{height}\kern0.1em -\kern0.1em 0.0217\times \mathrm{age}\kern0.1em -\kern0.1em 2.134\hfill \\ {}\mathrm{Predicted}\kern0.5em \mathrm{F}\mathrm{V}\mathrm{C}\left(\mathrm{L}\right):4.866\times \mathrm{height}\kern0.1em -\kern0.1em 0.020\times \mathrm{age}\kern0.1em -\kern0.1em 3.644\hfill \end{array} $$

FEV_1_ and FVC were then expressed as a percentage of the predicted values (FEV_1_%predicted and FVC%predicted). Sex-specific quartiles of FEV_1_%predicted and FVC%predicted were then constructed. In addition, we also tested the relationships with incidence of diabetes using published European prediction equations [23] in a sensitivity analysis.

Height (m) was measured using a fixed stadiometer; weight (kg) was measured using a balance beam scale. Blood samples were taken after an overnight fast and analysed using routine methods at the Department of Clinical Chemistry, Malmö University Hospital. ESR was determined according to the Westergren method. Information on smoking habits, alcohol use and use of anti-hypertensive medication or family history of diabetes was assessed in a questionnaire. Based on their responses to smoking habit questions, subjects were classified as never, former or current smokers. Alcohol abuse was assessed using a panel of nine questions related to alcohol use where >2 positive responses was considered problematic alcohol use [24]. In men, physical activity was assessed using a question *“Are you mostly engaged in sedentary activity in your spare time?”* Some questions were changed during the screening period. In women, physical activity was therefore assessed using the questions, *“Are you engaged in physical activity (e.g., swimming, gymnastics, badminton, tennis, folk dance, running, etc.) 1–2 hours per week?”* or *“Do you usually get to do light physical exercise like walking or cycling (or other activities with similar effort) on a regularly weekly basis?”*. Low socioeconomic status was defined as Statistics Sweden socioeconomic index (SEI) group 11–36 (i.e. unskilled or skilled manual workers or low-level non-manual workers).

Information about inflammatory markers was available in a subgroup of 5133 men. Complement 3 (C3), fibrinogen, ceruloplasmin, haptoglobin, orosomucoid and alpha-1 antitrypsin were analysed using electroimmunoassay. This subgroup has been described in detail elsewhere [25, 26].

### Endpoint ascertainment

Prevalent diabetes was defined as a fasting whole blood glucose ≥6.1 mmol/L at baseline (corresponding to a plasma glucose ≥7.0 mmol/L), self-reported diabetes or diabetes medication according to the questionnaire, or any prior diagnosis of diabetes in the registers used for follow-up (see below). Individuals with prevalent diabetes (*n* = 1173) were excluded from all analyses. All subjects were followed from the baseline examination until first diabetes event, death, emigration, or last follow-up date (31^st^ December 2012), whichever came first. Incident diabetes was defined using the Malmö HbA_1c_ Register (MHR), the Swedish National Diabetes Register (NDR), the Swedish Hospital Discharge Register, the Swedish Outpatient Register, the nationwide Swedish Drug Prescription register and the regional Diabetes 2000 Register of the Scania region. Incident cases of diabetes were also retrieved from re-examinations of individuals in the MPP cohort [19, 27]. The MHR register includes all glycated haemoglobin (HbA_1c_) values collected from individuals in institutional and non-institutional care in the greater Malmö area from 1988 onwards. HbA_1c_ was analysed at the Department of Clinical Chemistry, Malmö University Hospital. The Swedish Hospital discharge register has been operating in the south of Sweden since 1970 and became nationwide in 1987. The Swedish Drug Prescription register has been operating since 2005. NDR and the Diabetes 2000 Register required a physician diagnosis according to established diagnostic criteria (fasting plasma glucose concentration of ≥7.0 mmol/L measured on two different occasions). Individuals with at least two HbA_1c_ values ≥6.0 % according to the Swedish Mono-S standardization system (corresponding to 7.0 % with the US National Glycohemoglobin Standardization Program) recorded in the MHR after the baseline examination were defined as having incident diabetes mellitus (DM).

### Statistical analysis

All statistical analyses were carried out using SPSS version 22.0 and STATA V.12.0. To assess the relationship between baseline lung function and incident diabetes according to different follow-up times, the follow-up period was divided into four time intervals: 0–10 years, 10–20 years, 20–30 years and >30 years. Only the first diabetes events were counted, i.e. an individual could only be a diabetes case in one of the four time periods. The follow-up time was limited to the maximum time for each time interval. For example, for the 10–20 years interval, all participants with a follow-up time >10 years were included but only diabetes events with first diagnosis between 10 and 20 years were included in the analysis and any follow-up time over 20 years was limited to 20 years only. Sex-specific quartiles of FEV_1_%predicted and FVC%predicted were created (1 = low lung function and 4 = reference). Variables with a positively skewed distribution were log-transformed (ESR). One way analysis of variance (ANOVA) and Pearson’s chi-square test were used to compare baseline characteristics between subjects in quartiles of FEV_1_%predicted and FVC%predicted. Cox proportional hazards regression was used to assess incidence of diabetes according to different quartiles of lung function with and without adjustment for potential confounding factors. Cox proportional hazards assumption was tested visually using Kaplan Meier plots and log-log plots for the overall follow-up time. Fine and Gray’s proportional sub-hazards model was used to carry out competing risks regression to correct for the effect of deaths from causes unrelated to diabetes. Sub-hazard ratios for incident diabetes with deaths (with no diabetes diagnosis) as a competing event was obtained (adjusted for potential confounding factors).

## Results

Subject characteristics are presented in Tables 1 and 2. Those with poorer lung function reported a higher smoking prevalence in both men and women. Those in Q1 of FEV_1_%predicted had slightly higher BMI than those in Q4, and tended to be more physically inactive, have higher alcohol consumption and more likely to be on blood pressure (BP) medication. ESR and cholesterol levels were higher in Q1 vs. Q4 (*p* <0.001). Similar findings were seen for FVC%predicted (see Additional file 1: Table S1a and S1b).

**Table 1:** Baseline characteristics in relation to quartiles of FEV_1_%predicted: Males (*n* = 20,295)

	Overall	Q4	Q3	Q2	Q1	*P* value for trend
FEV_1_%predicted	95.4 (±17.7)	117.0 (±9.9)	100.7 (±3.1)	90.3 (±3.2)	73.3 (±11.3)	–
Number (*n*)	20,295	5074	5074	5074	5073	–
Age (years)	43.4 (±6.6)	43.9 (±6.5)	42.9 (±6.6)	42.8 (±6.6)	44.0 (±6.7)	0.350
Height (m)	1.77 (±0.07)	1.77 (±0.07)	1.77 (±0.07)	1.77 (±0.07)	1.77 (±0.07)	0.820
Current-smokers (%)	49.1	35.8	43.4	53.5	63.8	<0.001
BMI (kg/m^2^)	24.6 (±3.2)	24.4 (±2.9)	24.5 (±3.1)	24.5 (±3.2)	24.8 (±3.6)	<0.001
Physical inactivity (%)	52.4	45.9	50.4	54.6	58.6	<0.001
Anti-hypertensive medication (%)	3.7	3.0	3.5	3.5	4.7	<0.001
High alcohol consumption (%)	17.8	15.6	16.8	18.7	20.1	<0.001
ESR (mm/h)^a^	4.05	3.86	3.90	4.01	4.46	<0.001
Baseline glucose (mmol/L)	4.93 (±0.50)	4.89 (±0.49)	4.94 (±0.51)	4.94 (±0.50)	4.94 (±0.52)	<0.001
Cholesterol (mmol/L)	5.59 (±1.05)	5.52 (±1.01)	5.56 (±1.03)	5.60 (±1.06)	5.66 (±1.08)	<0.001
Family history of diabetes (%)	11.0	11.4	10.1	10.8	11.8	0.371
Social class (%)						0.125
- Low skilled	44.9	42.5	42.6	45.8	48.8	
- High skilled	43.9	47.1	47.3	42.3	38.8	
- Self-employed	8.3	8.0	8.0	9.0	8.0	
- Other	3.0	2.5	2.5	2.9	4.5	

*Q* Quartile, *FEV*_*1*_ Forced expiratory volume 1 s, *BMI* body mass index, *ESR* Erythrocyte sedimentation rate. Data consist of mean (±standard deviation) unless otherwise stated. ^a^Geometric mean presented for ESR. Linear by linear association for chi square tests used for *p* value for categorical variables, ANOVA test for linearity used for *p* value for continuous variables

**Table 2:** Baseline characteristics in relation to quartiles of FEV_1_%predicted: Females (*n* = 7416)

	Overall	Q4	Q3	Q2	Q1	*P* value for trend
FEV_1_%predicted	95.4 (±18.1)	116.9 (±10.2)	101.3 (±3.0)	90.9 (±3.2)	72.5 (±11.9)	–
Number (*n*)	7416	1854	1854	1854	1854	–
Age (years)	47.6 (±7.8)	48.0 (±6.6)	46.9 (±7.8)	46.6 (±8.6)	48.8 (±8.1)	0.023
Height (m)	1.64 (±0.06)	1.64 (±0.06)	1.64 (±0.06)	1.64 (±0.06)	1.64 (±0.06)	0.868
Current-smokers (%)	45.2	27.0	38.8	48.4	66.6	<0.001
BMI (kg/m^2^)	23.8 (±3.9)	23.8 (±3.5)	23.7 (±3.7)	23.7 (±3.9)	24.0 (±4.4)	0.135
Physical inactivity (%)	43.3	36.6	40.6	45.0	51.0	<0.001
- Missing data (%)	12.2	11.2	13.9	14.5	9.2	
Anti-hypertensive medication (%)	6.6	5.0	6.5	6.3	8.6	<0.001
High alcohol consumption (%)	2.7	2.5	2.4	2.9	3.1	0.150
ESR (mm/h)^a^	7.47	6.99	7.13	7.48	8.36	<0.001
Baseline glucose (mmol/L)	4.74 (±0.52)	4.70 (±0.52)	4.74 (±0.51)	4.75 (±0.53)	4.79 (±0.53)	<0.001
Cholesterol (mmol/L)	5.67 (±1.10)	5.64 (±1.07)	5.62 (±1.08)	5.62 (±1.11)	5.79 (±1.12)	<0.001
Family history of diabetes (%)	16.0	16.0	16.1	15.2	16.7	0.788
Social class (%)						0.785
- Low skilled	44.9	42.2	43.8	45.0	48.5	
- High skilled	45.4	48.5	48.2	45.4	39.5	
- Self-employed	2.9	3.1	2.3	3.2	3.0	
- Other	6.8	6.3	5.8	6.4	8.9	

*Q* Quartile, *FEV*_*1*_ Forced expiratory volume 1 s, *BMI* body mass index, *ESR* Erythrocyte sedimentation rate. Data consist of mean (±standard deviation) unless otherwise stated. ^a^Geometric mean presented for ESR. Linear by linear association for chi square tests used for *p* value for categorical variables, ANOVA test for linearity used for *p* value for continuous variables

### Incidence of diabetes

Mean follow-up was 27 years for men and 26 years for women. During full follow-up, there were 3753 and 993 incident diabetes events in men and women, respectively. The mean FEV_1_%predicted values for those who developed diabetes during follow-up and those who did not were 93.0 and 95.9 % respectively in men, and 92.6 and 95.8 % respectively in women. The corresponding values for FVC%predicted were 94.9 and 97.6 % respectively in men, and 94.8 and 97.6 % respectively in women. Hazard ratios (HR) of incident diabetes by quartiles of lung function are shown in Tables 3 and 4. For the overall follow-up time there was an approximately 40–50 % increase in adjusted risk of diabetes in Q1 of FEV_1_%predicted and FVC%predicted relative to Q4; for both males and females (see Additional file 1: Table S2a and S2b for FVC%predicted).

**Table 3:** Hazard ratios of diabetes mellitus by quartiles of FEV_1_%predicted in males: stratified by follow-up time (years) (*n* = 20,295)

follow-up time (years) (*n* = number of incident DM events)		Q4 (reference)	Q3	Q2	Q1	*P* value for trend
		≥106.34	95.57–106.34	84.65–95.57	≤84.65	
Overall follow-up time (*n* = 3753)	Unadjusted risk	1.00	1.13 (1.02–1.24)*	1.26 (1.15–1.39)***	1.77 (1.61–1.94)***	<0.001
	Adjusted risk^a^	1.00	1.06 (0.97–1.17)	1.15 (1.05–1.27)**	1.48 (1.35–1.63)***	<0.001
0–10 years (*n* = 365)	Unadjusted risk	1.00	1.09 (0.78–1.54)	1.25 (0.90–1.74)	2.41 (1.80–3.23)***	<0.001
	Adjusted risk^a^	1.00	0.99 (0.70–1.38	1.06 (0.76–1.47)	1.64 (1.21–2.22)**	<0.001
10–20 years (*n* = 1059)	Unadjusted risk	1.00	1.05 (0.87–1.27)	1.25 (1.04–1.50)*	1.94 (1.64–2.30)***	<0.001
	Adjusted risk^a^	1.00	0.98 (0.81–1.19)	1.12 (0.93–1.35)	1.52 (1.27–1.81)***	<0.001
20–30 years (*n* = 1984)	Unadjusted risk	1.00	1.18 (1.04–1.34)*	1.30 (1.15–1.48)***	1.59 (1.40–1.81)***	<0.001
	Adjusted risk*^a^	1.00	1.12 (0.98–1.27)	1.20 (1.05–1.36)**	1.39 (1.22–1.59)***	<0.001
>30 years (*n* = 345)	Unadjusted risk	1.00	1.08 (0.80–1.46)	1.10 (0.81–1.50)	1.59 (1.18–2.13)**	0.004
	Adjusted risk^a^	1.00	1.06 (0.79–1.44)	1.06 (0.77–1.44)	1.46 (1.08–1.97)*	0.023

^a^Adjusted for: age, height, BMI, smoking status, ESR (log transformed), baseline glucose, cholesterol, physical activity, BP medication, social class, family history of diabetes, and alcohol abuse. *p <0.05 **p < 0.01 ***p <0.001 *P* value for trend calculated using cox regression models (1 d.f)

**Table 4:** Hazard ratios of diabetes mellitus by quartiles of FEV_1_%predicted in females: stratified by follow-up time (years) (*n* = 7416)

follow-up time (years) (*n* = number of incident DM events)		Q4 (reference)	Q3	Q2	Q1	*P* value for trend
		≥106.67	96.13–106.67	85.14–96.12	≤85.13	
Overall follow-up time (*n* = 993)	Unadjusted risk	1.00	1.34 (1.11–1.62)**	1.40 (1.16–1.70)***	1.94 (1.62–2.33)***	<0.001
	Adjusted risk^a^	1.00	1.26 (1.04–1.53)*	1.26 (1.04–1.53)*	1.45 (1.20–1.75)***	<0.001
0–10 years (*n* = 135)	Unadjusted risk	1.00	2.19 (1.23–3.89)**	2.09 (1.17–3.72)*	2.77 (1.59–4.83)***	0.001
	Adjusted risk^a^	1.00	2.00 (1.12–3.58)*	1.72 (0.95–3.11)	1.68 (0.94–3.01)	0.275
10–20 years (*n* = 395)	Unadjusted risk	1.00	1.38 (1.01–1.87)*	1.44 (1.06–1.95)*	2.00 (1.50–2.67)***	<0.001
	Adjusted risk^a^	1.00	1.33 (0.98–1.81)	1.37 (1.01–1.87)*	1.51 (1.11–2.05)**	0.012
20–30 years (*n* = 419)	Unadjusted risk	1.00	1.13 (0.85–1.50)	1.33 (1.01–1.75)*	1.73 (1.32–2.28)***	<0.001
	Adjusted risk^a^	1.00	1.04 (0.78–1.39)	1.16 (0.87–1.54)	1.32 (0.99–1.76)	0.043
>30 years (*n* = 44)	Unadjusted risk	1.00	1.49 (0.60–3.69)	0.73 (0.26–2.00)	1.91 (0.78–4.70)	0.355
	Adjusted risk^a^	1.00	1.58 (0.63–3.94)	0.79 (0.28–2.20)	2.35 (0.93–5.90)	0.186

^a^Adjusted for: age, height, BMI, smoking status, ESR (log transformed), baseline glucose, cholesterol, physical activity, BP medication, social class, family history of diabetes, and alcohol abuse. *p <0.05 **p < 0.01 ***p < 0.001. *P* value for trend calculated using cox regression models (1 d.f)

HR for diabetes per 10 % decrease in FEV_1_%predicted for the overall follow-up time were 1.09 (CI: 1.07–1.11) in men, and 1.07 (CI: 1.03–1.11) in women. HR for diabetes per 10 % decrease in FVC%predicted were 1.10 (CI: 1.08–1.13) and 1.07 (CI: 1.03–1.11) in men and women, respectively.

We compared these results to those obtained from using European prediction equations for FEV_1_ and FVC [23] (see Additional file 1: Tables S3 and S4) and found the results to be very similar to the results from our own MPP cohort prediction equations.

The adjusted sub-hazards ratio, taking into account deaths from other causes before a diabetes diagnosis occurred, remained significant in Q1 and Q2 of both FEV_1_%predicted and FVC%predicted in men and women for the overall follow-up period (see Additional file 1: Table S5).

To assess the effect of inflammatory factors on the relationship between lung function and diabetes, a sub-analysis was carried out on a smaller cohort of men with information on ESR, white cell count (WCC), complement C3, fibrinogen, haptoglobin, ceruloplasmin, alpha-1 antitrypsin and orosomucoid (Table 5). For the overall follow-up period, HR (Q1 vs Q4) was 1.36 (CI: 1.14–1.63) for FEV_1_%predicted after adjusting for risk factors. The HR decreased slightly, but remained significant, after further adjustment for inflammatory markers (1.29, CI: 1.07–1.54, p for trend ≤0.001). HRs were slightly lower for FVC%predicted but largely similar (see Additional file 1: Table S6).

**Table 5:** Hazard ratios of diabetes mellitus by quartiles of FEV_1_%predicted in males: sub-cohort further adjusted for inflammation (5133 men)

follow-up time (years) (*n* = number of incident DM events)		Q4 (reference)	Q3	Q2	Q1	*P* value for trend
		≥106.39	95.20–106.39	83.51–95.17	≤83.50	
Follow-up time 0–37 years (*n* = 1025)	Adjusted risk^a^	1.00	0.94 (0.78–1.13)	1.24 (1.04–1.48)*	1.36 (1.14–1.63)**	<0.001
	Further adjusted for inflammation^b^	1.00	0.91 (0.75–1.09)	1.20 (1.01–1.44)*	1.29 (1.07–1.54)**	<0.001

^a^Adjusted for: age, height, BMI, smoking status, baseline glucose, cholesterol, physical activity, BP medication, social class, family history of diabetes, alcohol abuse ^b^Further adjusted for ESR (log transformed), WCC, Fibrinogen, complement C3, haptoglobin, ceruloplasmin, alpha-1 antitrypsin, and orosomucoid. *p <0.05 **p < 0.01. *P* value for trend calculated using cox regression models (1 d.f)

There was no significant interaction between smoking status and FEV_1_%predicted (*p* = 0.49 for men and 0.11 for women) or FVC%predicted (*p* = 0.88 for men and 0.98 for women) over the entire follow-up time (see Additional file 1: Table S7a-S7d for a stratified analysis by smoking status).

To assess the relationship between an obstructive lung pattern at baseline and future risk of diabetes we carried out an analysis comparing those with FEV_1_/FVC <70 % to those with FEV_1_/FVC ≥70 % (see Additional file 1: Table S8). After adjustment the risk for diabetes did not remain significant for both men and women with an FEV_1_/FVC <70 % relative to those ≥70 %.

Since diabetes can go undetected for a long time, an additional analysis was performed in a subgroup of 1530 men and women who were (a) re-screened with a measurement of fasting blood glucose as part of the Malmö diet and cancer (MDC-CC) study cohort and (b) still free from diabetes after more than 10 years (mean 14.1 years, range 10–18.4) from initial screening in the MPP study. A total of 214 subjects developed diabetes after this re-examination. In these subjects it was found that for every 10 % decrease in FEV_1_%predicted there was a 11 % increase in adjusted risk of diabetes (HR 1.11 (CI: 1.02–1.21)) and for every 10 % decrease in FVC%predicted there was a 12 % increase in adjusted risk of diabetes (HR 1.12 (CI: 1.02–1.23)). The corresponding HRs were significant also when standardised European reference values were used to calculate predicted FEV_1_ and FVC (HR 1.13 (CI: 1.03–1.23), and 1.14 (CI: 1.04–1.25), respectively).

### Incidence of diabetes by length of follow-up

**Fig. 2:**
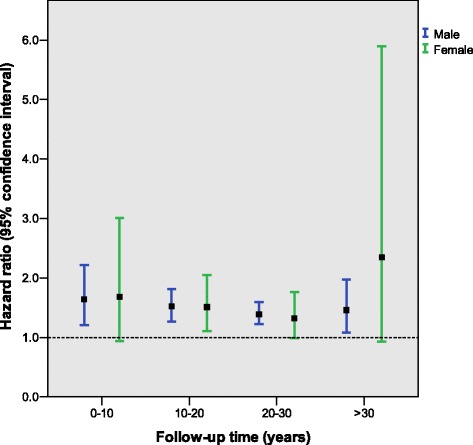
Hazard ratios for Q1 FEV_1_%predicted and incident diabetes for various follow-up times in males and females

When stratified into follow-up time intervals, low FEV_1_%predicted was found to be a significant risk factor for diabetes in men even after >30 years of follow-up (Fig. 2). The HR (Q4 vs. Q1) for diabetes was 1.64 (CI: 1.21–2.22) for events <10 years after baseline, 1.52 (CI: 1.27–1.81) for events 10–20 years after baseline, 1.39 (CI: 1.22–1.59) for events 20–30 years after baseline, and 1.46 (CI: 1.08–1.97) for events occurring >30 years after baseline. The relationships were largely similar for FVC%predicted (see Additional file 1: Table S2a and S2b) and for women, although confidence intervals were larger for women.

## Discussion

The temporal and causal relationship between lung function and diabetes has remained controversial in longitudinal studies. Our study explores the temporal relationship through stratified analyses based on follow-up time. The main finding was that the risk of future diabetes remains significant even after many years of follow-up from baseline poor lung function.

In our study of 27,711 subjects followed over 36–37 years, FVC and FEV_1_ were found to be powerful predictors of future diabetes, even following adjustment for baseline glucose levels and BMI. The mean baseline lung function was lower in those who then developed diabetes compared to those who did not. The differences were smaller than that reported previously [28]. However, since we report results from a longitudinal follow-up study of initially non-diabetic subjects, the results may differ from cross-sectional studies comparing diabetic cases and controls. In both men and women there was a high adjusted risk found even in non-smokers at certain time intervals (which was also higher than that of smokers during some time periods) confirming previous findings that the relationship of poor health outcomes associated with poor lung function goes beyond the effect of smoking [1, 29, 30]. As systemic inflammation has been thought to be a common cause of poor lung function and diabetes, we assessed this link using various inflammatory markers. Even following adjustment for these markers in a smaller cohort of men, there was still a 29 % increase in risk in Q1 of FEV_1_%predicted. Our study findings also show that an obstructive lung phenotype at baseline (i.e. low FEV_1_/FVC) is not associated with future diabetes risk (Additional file 1: Table S8), whereas poor %predicted values of either FEV_1_ or FVC continue to result in an increased diabetes risk even after many years duration. These findings are mirrored by other prospective studies where a reduction in FEV_1_ and FVC at baseline but not the ratio of FEV_1_/FVC is associated with an increased risk of diabetes [14, 17, 18].

There is a large body of studies that have assessed poor lung function in relation to diabetes or prediabetes risk [5, 10–18, 31–33]. There are substantial epidemiological implications of this association, in particular the possibility that diabetes and insulin resistance can explain the association between impaired lung function and the high risk of CVD and mortality [1, 34]. Our results have been in accordance with previous prospective studies that have assessed poor lung function and future risk of diabetes [5, 11, 14] and expand the results by exploring the increased risk over a long follow-up period.

The proposed mechanisms for the relationships between impaired lung function and diabetes include, for example, microangiopathy of lung vasculature, chronic inflammation, autonomic neuropathy, and loss of elastic recoil due to glycosylation of lung parenchyma, i.e., adverse effects of high glucose levels [9]. However, it seems unlikely that hyperglycaemia had major effects on lung function many years prior to the diagnosis of diabetes. Individuals with diabetes were excluded at baseline and in addition all results were adjusted for glucose at baseline. Our data suggest that there may exist a causal relationship between FEV_1_ and diabetes risk beyond the effects of hyperglycemia on lung function.

It has been proposed the mechanism that may link poor lung function to diabetes risk include reduced physical activity often associated with poor lung function, which predisposes to weight gain leading to the development of metabolic syndrome [13, 35]. In our study, risks were found to be significant even following adjustment for physical activity as assessed by questions based on the proportion of sedentary time in men and participation in high and low intensity physical activities in women. Chronic inflammation is also often thought to be a common factor linking lung function and diabetes risk. When adjusting for inflammatory factors in our study, there was a small reduction in diabetes risk which still remained significant for both Q1 of FEV_1_%predicted and FVC%predicted suggesting that markers of inflammation may not fully explain the underlying mechanism linking lung function and diabetes.

The role of early life factors can also play a vital role in the association between poor lung function and diabetes. Foetal programming and detrimental early life factors such as low birth weight can affect lung development and the development of diabetes, as it has been found that low birth weight is associated with poor adult lung function [36] and the later development of type 2 diabetes [37]. This hypothesis has been tested in a study by Yeh et al. [14] and it was found that although low FVC%predicted was associated with low birth weight, the association between FVC%predicted and incident diabetes was mainly independent of low birth weight. However further studies testing the role of foetal programming beyond the role of birth weight may be necessary, for example the influence of prematurity or maternal smoking during pregnancy.

Common genetic determinants for diabetes and poor lung function could be another possible explanation for the increased incidence of diabetes. Several genetic markers have been found to be associated with poor lung function [38]. However, the effects of single nucleotide polymorphisms are usually weak and it is unlikely that these markers explain more than a minor fraction of the relationship between poor lung function and risk of diabetes. Although this was beyond the scope of the present study, it nevertheless could be an aspect of future observational studies assessing the relationship between poor lung function and diabetes.

### Limitations

There are some methodological issues that need to be considered for this observational study. Firstly, the protocol was developed before the current guidelines for spirometry were developed. For example, no nose clips were used and only one acceptable manoeuvre of FEV_1_ and FVC was carried out. Although trained nursing staff were used to carry out measurements, this could potentially result in measurement errors. Due to the long follow-up time there may be some characteristics that may have changed during 36–37 years of follow-up such as smoking status, BMI or the use of medications such as statins and corticosteroids along with additional co-morbidities. The prevalence of smoking in Sweden decreased over the duration of this study [39] and we can assume that many smokers quit smoking during the follow-up. However, non-smokers that start smoking in adulthood are quite unusual [40], so we do not anticipate that the relationship between poor lung function and diabetes was confounded by an increase in smoking habits during the study. Statin treatment and the use of oral corticosteroids may indeed increase the risk of diabetes in some individuals, however where possible we have tried to control for many of these effects by adjustment for factors such cholesterol levels and BMI but there may be some residual effect of changes in these factors over time that could affect the outcome in some subjects.

It is well known that type 2 diabetes can go undetected for a long time and it is not possible to establish the exact time of onset. The definition of diabetes has changed over time and before 1998, when the World Health Organization (WHO) recommendations were published [41], plasma glucose ≥7.8 mmol/L was commonly used instead of the currently used cut-off of 7.0 mmol/L. This would increase incidence of diabetes during the last time periods. It is also possible that detection rates have increased due to increased awareness of diabetes in the health care organization. Also, the coverage of the registers used to identify new cases has improved over time, i.e. the first diagnosis of diabetes in our registers may not always represent the true onset of disease. However, in a subgroup analysis we found that reduced lung function was associated with future diabetes risk even in individuals whose blood glucose was normal more than 10 years (range 10–18 years) after the baseline examination. This strongly supports our conclusion that reduced lung function remains predictive for incident diabetes even after many years of follow-up.

Our findings were more statistically significant in men for trends across quartiles of both FEV_1_ and FVC%predicted and the risk of incident diabetes. However this can be due to statistical power as the population of women studied was much smaller. The point estimates were largely comparable in men and women.

We used various inflammatory markers to assess if a significant relationship between lung function and diabetes risk remains after taking systemic inflammation into account. Complement C3, WCC and fibrinogen have been associated with increased risk of diabetes and low lung function in previous studies [25, 26]. Although we included a wide range of inflammatory markers in the adjustment there may exist other important inflammatory markers, such as interleukin-6 (IL-6) and C-reactive Protein (CRP) [18, 42, 43], that may be associated with poor lung function and diabetes. Therefore the role of inflammation cannot be fully excluded. Obesity has been thought to be an important factor in the relationship between poor lung function and diabetes. We did not adjust our findings for abdominal obesity using waist circumference, therefore our measures of obesity corresponds to over overall obesity alone. Both BMI and waist circumference are major risk factors for type 2 diabetes and although waist circumference is a somewhat stronger predictor for type 2 diabetes compared to BMI, prospective studies show that the effects of these risk factors are largely comparable [44, 45].

Latent autoimmune diabetes in adults (LADA) is an additional important subgroup of adult diabetic subjects [46] and data on glutamic acid decarboxylase antibodies was not available for most cases. Even though a large majority of diabetes patients in this age group have Type 2 diabetes, and most cases with Type 1 diabetes were excluded from the study as prevalent cases, some incident cases were patients with LADA.

## Conclusion

Low FEV_1_ precedes and significantly predicts future diabetes, and the relationship is not fully explained by obesity, inflammation or smoking. This risk is still significant many years after the baseline FEV_1_ measurement in middle-aged men. These results suggest that there is a relationship between impaired lung function and diabetes risk beyond the specific effects of hyperglycemia on lung function. Further work is needed to establish if early life factors or genetic factors affecting both lung growth and impaired glucose metabolism may be responsible.

### Ethical approval and consent

The Health Service Authority of Malmö approved and funded the screening program. The linkage with the cause of death and patient registers was approved by the Regional ethics committee at Lund University. LU 85-2004; LU 2011-412. Written consent was not available at the time the study was conducted, however verbal consent was taken for all participants included in the study.

### Availability of data and materials

The MPP steering committee coordinates research using the MPP database. The data base is open for applications for research projects. Contact: Anders Dahlin, PhD, data manager, Email: Anders.Dahlin@med.lu.se.

## Additional file

Additional file 1: Tables S1a and S1b: Baseline characteristics in relation to quartiles of FVC%predicted, males and females respectively. Tables S2a and S2b: Hazard ratios of diabetes mellitus by quartiles of FVC%predicted, stratified by follow-up time, males and females respectively. Tables S3a and S3b: Hazard ratios of diabetes mellitus in males using MPP published equations and European reference equations for overall follow-up time, quartiles of FEV_1_%predicted and FVC%predicted respectively. Tables S4a and S4b: Hazard ratios of diabetes mellitus in females using MPP published equations and European reference equations for overall follow-up time, quartiles of FEV_1_%predicted and FVC%predicted respectively. Table S5: Sub-hazard ratios of diabetes mellitus by quartiles of FEV_1_%predicted and FVC%predicted in males and females for overall follow-up time. Table S6: Hazard ratios of diabetes mellitus by quartiles of FVC%predicted in males, sub-cohort further adjusted for inflammation. Tables S7a and S7b: Hazard ratios of diabetes mellitus by quartiles of FVC%predicted, stratified by follow-up time and smoking status, males and females respectively. Tables S7c and S7d: Hazard ratios of diabetes mellitus by quartiles of FEV_1_%predicted: Stratified by follow-up time and smoking status, males and females respectively. Table S8: Hazard ratios of diabetes mellitus by FEV1/FVC ratio, stratified by sex. (DOCX 72 kb)

## Abbreviations

ANOVA: one way analysis of variance
BMI: body mass index
CRP: C reactive protein
CVD: cardiovascular disease
DM: diabetes mellitus
ESR: erythrocyte sedimentation rate
FEV_1_: forced expiratory volume in 1 s
FVC: forced vital capacity
HbA_1C_: glycated haemoglobin
HR: hazard ratio
LADA: latent autoimmune diabetes in adults
MDC: Malmö Diet and Cancer Study
MDC-CC: Malmö Diet and Cancer Study: Cardiovascular Cohort
MHR: Malmö HbA_1C_ register
MPP: Malmö Preventive Project
NDR: Swedish National Diabetes Register
Q: quartile
WCC: white cell count
WHO: World Health Organization
