# Supplementary material for: The temporal relationship between poor lung function and the risk of diabetes
Source: BMC Pulm Med. 2016 May 10;16:75. doi: 10.1186/s12890-016-0227-z (PMC4863358; doi:10.1186/s12890-016-0227-z)
Supplement: Additional file 1: — Tables S1a and S1b: Baseline characteristics in relation to quartiles of FVC%predicted, males and females respectively. Tables S2a and S2b: Hazard ratios of diabetes mellitus by quartiles of FVC%predicted, stratified by follow-up time, males and females respectively. Tables S3a and S3b: Hazard ratios of diabetes mellitus in males using MPP published equations and European reference equations for overall follow-up time, quartiles of FEV1%predicted and FVC%predicted respectively. Tables S4a and S4b: Hazard ratios of diabetes mellitus in females using MPP published equations and European reference equations for overall follow-up time, quartiles of FEV1%predicted and FVC%predicted respectively. Table S5: Sub-hazard ratios of diabetes mellitus by quartiles of FEV1%predicted and FVC%predicted in males and females for overall follow-up time. Table S6: Hazard ratios of diabetes mellitus by quartiles of FVC%predicted in males, sub-cohort further adjusted for inflammation. Tables S7a and S7b: Hazard ratios of diabetes mellitus by quartiles of FVC%predicted, stratified by follow-up time and smoking status, males and females respectively. Tables S7c and S7d: Hazard ratios of diabetes mellitus by quartiles of FEV1%predicted: Stratified by follow-up time and smoking status, males and females respectively. Table S8: Hazard ratios of diabetes mellitus by FEV1/FVC ratio, stratified by sex. (DOCX 72 kb) [file 12890_2016_227_MOESM1_ESM.docx]

**Table S1a: Baseline characteristics in relation to quartiles of FVC%predicted: Males (n=20295)**

|  | **Overall** | **Q4** | **Q3** | **Q2** | **Q1** | **P value for trend** |
| --- | --- | --- | --- | --- | --- | --- |
| FVC %predicted | 97.1 (±16.2) | 117.0 (±9.5) | 101.9 (±2.7) | 92.7 (±2.9) | 76.8 (±9.8) | - |
| Number (n) | 20295 | 5074 | 5074 | 5074 | 5073 | - |
| Age (years) | 43.4 (±6.6) | 43.8 (±6.3) | 43.0 (±6.6) | 42.7 (±6.7) | 44.0 (±6.9) | 0.449 |
| Height (m) | 1.77 (±0.07) | 1.77 (±0.07) | 1.77 (±0.07) | 1.78 (±0.07) | 1.77 (±0.07) | <0.001 |
| Current-smokers (%) | 49.1 | 40.1 | 45.0 | 51.2 | 60.2 | <0.001 |
| BMI (kg/m^2^) | 24.6 (±3.2) | 24.4 (±2.9) | 24.4 (±3.0) | 24.5 (±3.3) | 24.9 (±3.6) | <0.001 |
| Physical inactivity (%) | 52.4 | 45.5 | 51.0 | 54.4 | 58.5 | <0.001 |
| Anti-hypertensive medication (%) | 3.7 | 3.0 | 3.2 | 3.7 | 4.8 | <0.001 |
| High alcohol consumption (%) | 17.8 | 15.1 | 17.8 | 18.7 | 19.5 | <0.001 |
| ESR (mm/hr)* | 4.05 | 3.91 | 3.87 | 4.00 | 4.45 | <0.001 |
| Baseline glucose (mmol/L) | 4.93 (±0.50) | 4.89 (±0.50) | 4.93 (±0.50) | 4.95 (±0.50) | 4.93 (±0.52) | <0.001 |
| Cholesterol (mmol/L) | 5.59 (±1.05) | 5.53(±1.02) | 5.58 (±1.02) | 5.58 (±1.07) | 5.66 (±1.08) | <0.001 |
| Family history of diabetes (%) | 11.0 | 11.4 | 10.5 | 10.7 | 11.5 | 0.784 |
| Social class (%)  - Low skilled  - High skilled  - Self-employed  - Other | 44.9  43.9  8.3  3.0 | 43.4  47.1  7.2  2.4 | 42.5  46.1  9.1  2.3 | 45.6  43.4  8.4  2.5 | 48.1  38.9  8.4  4.6 | 0.860 |

Q: Quartile; FVC: Forced vital capacity; BMI: body mass index; ESR: Erythrocyte sedimentation rate. *Geometric mean presented for ESR. Data consists of mean (±standard deviation) unless otherwise stated. Linear by linear association for chi square tests used for p value for categorical variables, ANOVA test for linearity used for p value for continuous variables.

**Table S1b : Baseline characteristics in relation to quartiles of FVC%predicted: Females (n=7416)**

|  | **Overall** | **Q4** | **Q3** | **Q2** | **Q1** | **P value for trend** |
| --- | --- | --- | --- | --- | --- | --- |
| FVC %predicted | 97.3 (±17.0) | 117.8(±10.2) | 102.1 (±2.9) | 93.0 (±2.6) | 76.1 (±10.9) | - |
| Number (n) | 7416 | 1854 | 1854 | 1854 | 1854 | - |
| Age (years) | 47.6 (±7.8) | 48.0 (±6.7) | 47.4 (±7.5) | 46.8 (±8.3) | 48.1 (±8.7) | 0.449 |
| Height (m) | 1.64 (±0.06) | 1.64 (±0.06) | 1.64 (±0.06) | 1.64 (±0.06) | 1.64 (±0.06) | 0.402 |
| Current-smokers (%) | 45.2 | 33.0 | 38.1 | 48.3 | 61.3 | <0.001 |
| BMI (kg/m^2^) | 23.8 (±3.9) | 23.8 (±3.5) | 23.7 (±3.7) | 23.8 (±4.0) | 23.9 (±4.4) | 0.405 |
| Physical inactivity (%)   - Missing data | 43.3  12.2 | 37.2  14.1 | 40.0  12.9 | 44.1  13.2 | 51.9  8.5 | 0.070 |
| Anti-hypertensive medication (%) | 6.6 | 5.4 | 5.5 | 6.3 | 9.2 | <0.001 |
| High alcohol consumption (%) | 2.7 | 2.7 | 2.2 | 2.6 | 3.3 | 0.190 |
| ESR (mm/hr)* | 7.47 | 7.13 | 7.15 | 7.52 | 8.14 | <0.001 |
| Baseline glucose (mmol/L) | 4.74 (±0.52) | 4.72 (±0.51) | 4.73 (±0.52) | 4.74 (±0.53) | 4.79 (±0.53) | <0.001 |
| Cholesterol (mmol/L) | 5.67 (±1.10) | 5.63 (±1.03) | 5.65 (±1.09) | 5.62 (±1.10) | 5.76 (±1.15) | 0.002 |
| Family history of diabetes (%) | 16.0 | 16.8 | 15.4 | 15.6 | 16.1 | 0.620 |
| Social class (%)  - Low skilled  - High skilled  - Self-employed  - Other | 44.9  45.4  2.9  6.8 | 44.1  47.3  2.8  5.9 | 42.7  48.3  2.8  6.3 | 44.7  45.9  2.9  6.6 | 48.1  40.1  3.2  8.6 | 0.573 |

Q: Quartile; FVC: Forced vital capacity; BMI: body mass index; ESR: Erythrocyte sedimentation rate *Geometric mean presented for ESR. Data consists of mean (±standard deviation) unless otherwise stated. Linear by linear association for chi square tests used for p value for categorical variables, ANOVA test for linearity used for p value for continuous variables.

**Table S2a: Hazard ratios of diabetes mellitus by quartiles of FVC%predicted in males: Stratified by follow up time (years) (n= 20 295)**

| **Follow up time (years)**  **(n= number of incident DM events)** |  | **Q4**  **(reference)** | **Q3** | **Q2** | **Q1** | **P value for trend** |
| --- | --- | --- | --- | --- | --- | --- |
|  |  | ≥106.88 | 97.42-106.88 | 87.34-97.42 | ≤87.34 |  |
|  |  |  |  |  |  |  |
| Overall follow-up time (n=3753) | Unadjusted risk | 1.00 | 1.10 (1.00-1.22)* | 1.32 (1.20-1.45)*** | 1.76 (1.60-1.92)*** | <0.001 |
|  | Adjusted risk† | 1.00 | 1.06 (0.96-1.17) | 1.24 (1.13-1.37)*** | 1.48 (1.35-1.63)*** | <0.001 |
|  |  |  |  |  |  |  |
| 0-10 years (n= 365) | Unadjusted risk | 1.00 | 1.06 (0.76-1.50) | 1.41 (1.02-1.94)* | 2.28 (1.70-3.06)*** | <0.001 |
|  | Adjusted risk† | 1.00 | 1.01 (0.72-1.43) | 1.21 (0.88-1.68) | 1.56 (1.15-2.18)** | 0.001 |
|  |  |  |  |  |  |  |
|  |  |  |  |  |  |  |
| 10-20 years (n= 1059) | Unadjusted risk | 1.00 | 1.08 (0.89-1.31) | 1.41 (1.17-1.69)*** | 2.01 (1.69-2.38)*** | <0.001 |
|  | Adjusted risk† | 1.00 | 1.04 (0.86-1.27) | 1.29 (1.07-1.55)** | 1.57 (1.32-1.87)*** | <0.001 |
|  |  |  |  |  |  |  |
|  |  |  |  |  |  |  |
| 20-30 years (n=1984) | Unadjusted risk | 1.00 | 1.09 (0.96-1.24) | 1.30 (1.15-1.48)*** | 1.64 (1.45-1.86)*** | <0.001 |
|  | Adjusted risk† | 1.00 | 1.04 (0.91-1.19) | 1.24 (1.09-1.41)** | 1.45 (1.28-1.65)*** | <0.001 |
|  |  |  |  |  |  |  |
|  |  |  |  |  |  |  |
| >30 years (n= 345) | Unadjusted risk | 1.00 | 1.25 (0.94-1.67) | 1.14 (0.85-1.55) | 1.19 (0.87-1.63) | 0.383 |
|  | Adjusted risk† | 1.00 | 1.22 (0.92-1.64) | 1.13 (0.83-1.53) | 1.09 (0.79-1.50) | 0.716 |
|  |  |  |  |  |  |  |

†Adjusted for: age, height, BMI, smoking status, ESR (log transformed), baseline glucose, cholesterol, physical activity, BP medication, social class, family history of diabetes, and alcohol abuse. *p <0.05 **p<0.01 ***p <0.001. P value for trend calculated using cox regression models (1 d.f).

**Table S2b: Hazard ratios of diabetes mellitus by quartiles of FVC%predicted in females: Stratified by follow up time (years) (n= 7416)**

| **Follow up time (years)**  **(n= number of incident DM events)** |  | **Q4**  **(reference)** | **Q3** | **Q2** | **Q1** | **P value for trend** |
| --- | --- | --- | --- | --- | --- | --- |
|  |  | ≥107.50 | 97.33-107.49 | 87.95-97.30 | ≤87.94 |  |
|  |  |  |  |  |  |  |
| Overall follow-up time (n=993) | Unadjusted risk | 1.00 | 1.06 (0.87-1.28) | 1.35 (1.12-1.62)** | 1.75 (1.46-2.09)*** | <0.001 |
|  | Adjusted risk† | 1.00 | 1.03 (0.85-1.24) | 1.31 (1.09-1.58)** | 1.39 (1.16-1.67)*** | <0.001 |
|  |  |  |  |  |  |  |
| 0-10 years (n=135 ) | Unadjusted risk | 1.00 | 1.89 (1.07-3.35)* | 2.07 (1.18-3.64)* | 2.60 (1.51-4.49)** | 0.001 |
|  | Adjusted risk† | 1.00 | 1.75 (0.98-3.11) | 1.70 (0.96-3.01) | 1.55 (0.88-2.71) | 0.269 |
|  |  |  |  |  |  |  |
|  |  |  |  |  |  |  |
| 10-20 years (n=395 ) | Unadjusted risk | 1.00 | 1.09 (0.80-1.48) | 1.39 (1.04-1.87)* | 1.90 (1.43-2.51)*** | <0.001 |
|  | Adjusted risk† | 1.00 | 1.07 (0.79-1.46) | 1.40 (1.04-1.88)* | 1.50 (1.12-2.00)** | 0.002 |
|  |  |  |  |  |  |  |
|  |  |  |  |  |  |  |
| 20-30 years (n=419) | Unadjusted risk | 1.00 | 0.92 (0.69-1.22) | 1.19 (0.90-1.55) | 1.54 (1.18-2.02)** | <0.001 |
|  | Adjusted risk† | 1.00 | 0.89 (0.67-1.19) | 1.16 (0.88-1.52) | 1.28 (0.97-1.69) | 0.026 |
|  |  |  |  |  |  |  |
|  |  |  |  |  |  |  |
| >30 years (n=44 ) | Unadjusted risk | 1.00 | 0.71 (0.28-1.79) | 1.21 (0.54-2.68) | 1.07 (0.45-2.52) | 0.577 |
|  | Adjusted risk† | 1.00 | 0.69 (0.27-1.77) | 1.39 (0.61-3.16) | 1.28 (0.52-3.12) | 0.301 |
|  |  |  |  |  |  |  |

†Adjusted for: age, height, BMI, smoking status, ESR (log transformed), baseline glucose, cholesterol, physical activity, BP medication, social class, family history of diabetes, and alcohol abuse. *p <0.05 **p<0.01 ***p <0.001. P value for trend calculated using cox regression models (1 d.f).

**Table S3a: Hazard ratios of diabetes mellitus by quartiles of FEV_1_%predicted in males using MPP published equations and European reference equations for overall follow-up time (n= 20 295)**

|  |  | Q4 | Q3 | Q2 | Q1 | P value trend |
| --- | --- | --- | --- | --- | --- | --- |
|  | MPP cohort | ≥106.34 | 95.57-106.34 | 84.65-95.57 | ≤84.65 |  |
|  | European | ≥101.59 | 91.37-101.58 | 80.79-91.37 | ≤80.79 |  |
|  |  |  |  |  |  |  |
| **MPP cohort equation** | Unadjusted risk | 1.00 | 1.13 (1.02-1.24)* | 1.26 (1.15-1.39)*** | 1.77 (1.61-1.94)*** | <0.001 |
| **European equation** | Unadjusted risk | 1.00 | 1.18 (1.07-1.29)** | 1.32 (1.20-1.45)*** | 1.84 (1.68-2.02)*** | <0.001 |
| **MPP cohort equation** | Adjusted* risk | 1.00 | 1.06 (0.97-1.17) | 1.15 (1.05-1.27)** | 1.48 (1.35-1.63)*** | <0.001 |
| **European equation** | Adjusted* risk | 1.00 | 1.09 (0.99-1.20) | 1.17 (1.06-1.28)** | 1.46 (1.33-1.60)*** | <0.001 |

Adjusted for: age, height, BMI, smoking status, ESR (log transformed), baseline glucose, cholesterol, physical activity, BP medication, social class, family history of diabetes, and alcohol abuse. *p <0.05 **p<0.01 ***p <0.001. P value for trend calculated using cox regression models (1 d.f). MPP: Malmö Preventive Project.

**Table S3b: Hazard ratios of diabetes mellitus by quartiles of FVC%predicted in males using MPP published equations and European reference equations for overall follow-up time (n= 20 295)**

|  |  | Q4 | Q3 | Q2 | Q1 | P value trend |
| --- | --- | --- | --- | --- | --- | --- |
|  | MPP cohort | ≥106.88 | 97.42-106.88 | 87.34-97.42 | ≤87.34 |  |
|  | European | ≥105.59 | 96.44-105.59 | 86.17-96.44 | ≤86.17 |  |
|  |  |  |  |  |  |  |
| **MPP cohort equation** | Unadjusted risk | 1.00 | 1.10 (1.00-1.22)* | 1.32 (1.20-1.45)*** | 1.76 (1.60-1.92)*** | <0.001 |
| **European equation** | Unadjusted risk | 1.00 | 1.12 (1.01-1.23)* | 1.36 (1.23-1.49)*** | 1.82 (1.66-1.99)*** | <0.001 |
| **MPP cohort equation** | Adjusted* risk | 1.00 | 1.06 (0.96-1.17) | 1.24 (1.13-1.37)*** | 1.48 (1.35-1.63)*** | <0.001 |
| **European equation** | Adjusted risk* | 1.00 | 1.06 (0.96-1.17) | 1.21 (1.10-1.33)*** | 1.47 (1.34-1.62)*** | <0.001 |

Adjusted for: age, height, BMI, smoking status, ESR (log transformed), baseline glucose, cholesterol, physical activity, BP medication, social class, family history of diabetes, and alcohol abuse. *p <0.05 **p<0.01 ***p <0.001. P value for trend calculated using cox regression models (1 d.f). MPP: Malmö Preventive Project.

**Table S4a: Hazard ratios of diabetes mellitus by quartiles of FEV_1_%predicted in females using MPP published equations and European reference equations for overall follow-up time (n= 7416)**

|  |  | Q4 | Q3 | Q2 | Q1 | P value trend |
| --- | --- | --- | --- | --- | --- | --- |
|  | MPP cohort | ≥106.67 | 96.13-106.67 | 85.14-96.12 | ≤85.13 |  |
|  | European | ≥109.86 | 98.71-109.86 | 87.48-98.71 | ≤87.48 |  |
|  |  |  |  |  |  |  |
| **MPP cohort equation** | Unadjusted risk | 1.00 | 1.34 (1.11-1.62)** | 1.40 (1.16-1.70)*** | 1.94 (1.62-2.33)*** | <0.001 |
| **European equation** | Unadjusted risk | 1.00 | 1.21 (1.00-1.46) | 1.31(1.09-1.57)** | 1.76 (1.47-2.10)*** | <0.001 |
| **MPP cohort equation** | Adjusted risk* | 1.00 | 1.26 (1.04-1.53)* | 1.26 (1.04-1.53)* | 1.45 (1.20-1.75)*** | <0.001 |
| **European equation** | Adjusted risk* | 1.00 | 1.19 (0.98-1.44) | 1.28 (1.06-1.55)* | 1.41 (1.17-1.70)*** | <0.001 |

Adjusted for: age, height, BMI, smoking status, ESR (log transformed), baseline glucose, cholesterol, physical activity, BP medication, social class, family history of diabetes, and alcohol abuse. *p <0.05 **p<0.01 ***p <0.001. P value for trend calculated using cox regression models (1 d.f). MPP: Malmö Preventive Project.

**Table S4b: Hazard ratios of diabetes mellitus by quartiles of FVC%predicted in females using MPP published equations and European reference equations for overall follow-up time: (n= 7416)**

|  |  | Q4 | Q3 | Q2 | Q1 | P value trend |
| --- | --- | --- | --- | --- | --- | --- |
|  | MPP cohort | ≥107.50 | 97.33-107.49 | 87.95-97.30 | ≤87.94 |  |
|  | European | ≥116.32 | 105.02-116.32 | 94.59-105.01 | ≤94.59 |  |
|  |  |  |  |  |  |  |
| **MPP cohort equation** | Unadjusted risk | 1.00 | 1.06 (0.87-1.28) | 1.35 (1.12-1.62)** | 1.75 (1.46-2.09)*** | <0.001 |
| **European equation** | Unadjusted risk | 1.00 | 1.11 (0.92-1.34) | 1.36 (1.13-1.63)** | 1.66 (1.39-1.99)*** | <0.001 |
| **MPP cohort equation** | Adjusted risk* | 1.00 | 1.03 (0.85-1.24) | 1.31 (1.09-1.58)** | 1.39 (1.16-1.67)*** | <0.001 |
| **European equation** | Adjusted risk* | 1.00 | 1.10 (0.91-1.33) | 1.33 (1.11-1.60)** | 1.47 (1.22-1.76)*** | <0.001 |

Adjusted for: age, height, BMI, smoking status, ESR (log transformed), baseline glucose, cholesterol, physical activity, BP medication, social class, family history of diabetes, and alcohol abuse. *p <0.05 **p<0.01 ***p <0.001. P value for trend calculated using cox regression models (1 d.f). MPP: Malmö Preventive Project.

**Table S5: Sub-hazard ratios of diabetes mellitus by quartiles of FEV_1_%predicted and FVC %predicted in males and females for overall follow-up time: (n= 20 295 males and 7416 females)**

| **Follow up time (years)**  **(n= number of incident DM events)** |  | **Q4**  **(reference)** | **Q3** | **Q2** | **Q1** | **P value for trend** |
| --- | --- | --- | --- | --- | --- | --- |
| **FEV_1_%predicted males** |  | **≥106.34** | **95.57-106.34** | **84.65-95.57** | **≤84.65** |  |
| Competing risks regression for overall follow-up time  (n=3753) | Adjusted risk | 1.00 | 1.04 (0.95-1.15) | 1.13 (1.03-1.25)* | 1.33 (1.21-1.47)*** | <0.001 |
| **FEV_1_%predicted females** |  | **≥106.67** | **96.13-106.67** | **85.14-96.12** | **≤85.13** |  |
| Competing risks regression for overall follow-up time (n=993) | Adjusted risk | 1.00 | 1.25 (1.03-1.51)* | 1.23 (1.01-1.50)* | 1.31 (1.08-1.60)** | 0.016 |
| **FVC %predicted males** |  | **≥106.88** | **97.42-106.88** | **87.34-97.42** | **≤87.34** |  |
| Competing risks regression for overall follow-up time (n=3753) | Adjusted risk | 1.00 | 1.06 (0.96-1.17) | 1.22 (1.10-1.34)*** | 1.35 (1.23-1.48)*** | <0.001 |
| **FVC %predicted females** |  | **≥107.50** | **97.33-107.49** | **87.95-97.30** | **≤87.94** |  |
| Competing risks regression for overall follow-up time (n=993) | Adjusted risk | 1.00 | 1.02 (0.84-1.24) | 1.28 (1.06-1.53)* | 1.27 (1.05-1.53)* | 0.003 |

Adjusted for: age, height, BMI, smoking status, ESR (log transformed), baseline glucose, cholesterol, physical activity, BP medication, social class, family history of diabetes, and alcohol abuse. *p <0.05 **p<0.01 ***p <0.001. P value for trend calculated using cox regression models (1 d.f). Competing risks regression; 3753 incident diabetes cases as failure event and 6609 deaths without diabetes as competing risk in males and 993 incident diabetes cases as failure event and 1746 deaths without diabetes as competing risk in females

**Table S6: Hazard ratios of diabetes mellitus by quartiles of FVC%predicted in males: Sub-cohort further adjusted for inflammation (5133 men)**

| **Follow up time (years)**  **(n= number of incident DM events)** |  | **Q4**  **(reference)** | | **Q3** | | **Q2** | | **Q1** | **P value for trend** |
| --- | --- | --- | --- | --- | --- | --- | --- | --- | --- |
|  |  | ≥107.55 | | 97.34-107.54 | | 86.70-97.33 | | ≤86.70 |  |
|  |  |  | |  | |  | |  |  |
| Follow-up time 0-37 years (n=1025) | Adjusted risk† | 1.00 | | 0.95 (0.79-1.14) | | 1.33 (1.11-1.58)** | | 1.26 (1.05-1.51)* | <0.001 |
|  | Further adjusted for inflammation†† | 1.00 | | 0.93 (0.77-1.12) | | 1.32 (1.10-1.57)** | | 1.21 (1.01-1.46)* | 0.001 |
|  |  |  |  | |  | |  | |  |

†Adjusted for: age, height, BMI, smoking status, baseline glucose, cholesterol, physical activity, BP medication, social class, family history of diabetes, alcohol abuse. †† Further adjusted for ESR (log transformed), WBC, Fibrinogen, complement C3, haptoglobin, ceruloplasmin, alpha-1 antitrypsin and orosomucoid. *p <0.05 **p<0.01. P value for trend calculated using cox regression models (1 d.f)

**Table S7a Hazard ratios of diabetes mellitus by quartiles of FVC %predicted in males: Stratified by follow up time and smoking status (10324 non-smokers and 9971 smokers)**

| **Follow up time (years)**  **(n= number of incident DM events)** |  |  | **Q4**  **(reference)** | **Q3** | **Q2** | **Q1** | **P value for trend** |
| --- | --- | --- | --- | --- | --- | --- | --- |
| **Non-smokers** |  |  | ≥108.85 | 99.56-108.85 | 90.15-99.56 | ≤90.15 |  |
| **Smokers** |  |  | ≥104.49 | 94.96-104.47 | 84.58-94.96 | ≤84.58 |  |
|  |  |  |  |  |  |  |  |
| 0-10 years  Non-smokers (n=169)  Smokers (n=196) | Unadjusted risk | Non-smokers | 1.00 | 0.87 (0.53-1.45) | 1.25 (0.79-1.99) | 2.17 (1.43-3.30)*** | <0.001 |
|  |  | Smokers | 1.00 | 1.17 (0.74-1.83) | 1.35 (0.82-2.09) | 2.13 (1.43-3.19)*** | <0.001 |
|  | Adjusted risk† | Non-smokers | 1.00 | 0.83 (0.50-1.39) | 1.11 (0.69-1.79) | 1.55 (1.01-2.38)* | 0.012 |
|  |  | Smokers | 1.00 | 1.05 (0.66-1.65) | 1.15 (0.74-1.78) | 1.45 (0.96-2.19) | 0.052 |
|  |  |  |  |  |  |  |  |
| 10-20 years  Non-smokers (n=482)  Smokers (n=577) | Unadjusted risk | Non-smokers | 1.00 | 0.95 (0.72-1.25) | 1.04 (0.80-1.36) | 1.69 (1.32-2.15)*** | <0.001 |
|  |  | Smokers | 1.00 | 1.36 (1.04-1.77)* | 1.77 (1.37-2.28)*** | 2.25 (1.76-2.87)*** | <0.001 |
|  | Adjusted risk† | Non-smokers | 1.00 | 0.95 (0.72-1.25) | 0.98 (0.75-1.28) | 1.37 (1.07-1.76)* | 0.007 |
|  |  | Smokers | 1.00 | 1.33 (1.02-1.74)* | 1.65 (1.28-2.13)*** | 1.89 (1.47-2.42)*** | <0.001 |
|  |  |  |  |  |  |  |  |
| 20-30 years  Non-smokers (n=1001)  Smokers (n=983 ) | Unadjusted risk | Non-smokers | 1.00 | 1.23 (1.02-1.48)* | 1.30 (1.08-1.57)** | 1.79 (1.50-2.14)*** | <0.001 |
|  |  | Smokers | 1.00 | 1.04 (0.87-1.25) | 1.20 (1.01-1.44)* | 1.39 (1.16-1.66)*** | <0.001 |
|  | Adjusted risk† | Non-smokers | 1.00 | 1.19 (0.99-1.44) | 1.25 (1.04-1.51)* | 1.64 (1.37-1.97)*** | <0.001 |
|  |  | Smokers | 1.00 | 1.02 (0.85-1.22) | 1.15 (0.96-1.37) | 1.27 (1.06-1.52)* | 0.004 |
|  |  |  |  |  |  |  |  |
| >30 years  Non-smokers (n=180)  Smokers (n=165 ) | Unadjusted risk | Non-smokers | 1.00 | 0.86 (0.57-1.28) | 0.93 (0.63-1.39) | 0.86 (0.57-1.31) | 0.572 |
|  |  | Smokers | 1.00 | 0.99 (0.65-1.50) | 1.01 (0.66-1.56) | 1.12 (0.72-1.75) | 0.618 |
|  | Adjusted risk† | Non-smokers | 1.00 | 0.84 (0.56-1.26) | 0.93 (0.62-1.38) | 0.79 (0.52-1.21) | 0.375 |
|  |  | Smokers | 1.00 | 1.01 (0.66-1.53) | 1.04 (0.68-1.60) | 1.10 (0.71-1.72) | 0.659 |

†Adjusted for: age, height, BMI, ESR (log transformed), baseline glucose, cholesterol, physical activity, BP medication, social class, family history of diabetes, and alcohol abuse. *p <0.05 **p<0.01 ***p <0.001. P value for trend calculated using cox regression models(1 d.f)

**Table S7b Hazard ratios of diabetes mellitus by quartiles of FVC %predicted in females: Stratified by follow up time and smoking status (4065 non-smokers, 3351 smokers)**

| **Follow up time (years)**  **(n= number of incident DM events)** |  |  | **Q4**  **(reference)** | **Q3** | **Q2** | **Q1** | **P value for trend** |
| --- | --- | --- | --- | --- | --- | --- | --- |
| **Non-smokers** |  |  | ≥110.18 | 100.30-110.16 | 91.68-100.30 | ≤91.68 |  |
| **Smokers** |  |  | ≥103.75 | 93.80-103.75 | 83.42-93.79 | ≤83.39 |  |
|  |  |  |  |  |  |  |  |
| 0-10 years  Non-smokers (n=62)  Smokers (n=73) | Unadjusted risk | Non-smokers | 1.00 | 2.44 (1.01-5.89)* | 2.01 (0.81-4.97) | 3.47 (1.50-8.06)** | 0.007 |
|  |  | Smokers | 1.00 | 2.00 (0.93-4.26) | 2.33 (1.11-4.89)* | 2.04 (0.95-4.35) | 0.074 |
|  | Adjusted risk† | Non-smokers | 1.00 | 2.41 (0.99-5.87) | 1.87 (0.75-4.69) | 2.25 (0.95-5.32) | 0.163 |
|  |  | Smokers | 1.00 | 1.63 (0.75-3.53) | 1.75 (0.82-3.73) | 1.20 (0.55-2.60) | 0.848 |
|  |  |  |  |  |  |  |  |
| 10-20 years  Non-smokers (n=204)  Smokers (n=191) | Unadjusted risk | Non-smokers | 1.00 | 1.32 (0.87-2.00) | 1.17 (0.76-1.80) | 1.87 (1.26-2.76)** | 0.004 |
|  |  | Smokers | 1.00 | 1.35 (0.86-2.13) | 2.12 (1.39-3.22)*** | 1.72 (1.11-2.66)* | 0.003 |
|  | Adjusted risk† | Non-smokers | 1.00 | 1.29 (0.85-1.96) | 1.09 (0.71-1.67) | 1.58 (1.06-2.36)* | 0.050 |
|  |  | Smokers | 1.00 | 1.33 (0.84-2.10) | 2.02 (1.32-3.08)** | 1.30 (0.83-2.03) | 0.117 |
|  |  |  |  |  |  |  |  |
| 20-30 years  Non-smokers (n=227)  Smokers (n=192) | Unadjusted risk | Non-smokers | 1.00 | 0.86 (0.58-1.27) | 1.17 (0.82-1.69) | 1.30 (0.91-1.87) | 0.062 |
|  |  | Smokers | 1.00 | 0.77 (0.51-1.17) | 1.02 (0.69-1.52) | 1.49 (1.02-2.19)* | 0.023 |
|  | Adjusted risk† | Non-smokers | 1.00 | 0.87 (0.58-1.29) | 1.01 (0.70-1.46) | 1.22 (0.85-1.77) | 0.211 |
|  |  | Smokers | 1.00 | 0.76 (0.50-1.15) | 0.97 (0.65-1.45) | 1.24 (0.84-1.84) | 0.175 |
|  |  |  |  |  |  |  |  |
| >30 years  Non-smokers (n=28)  Smokers (n=16) | Unadjusted risk | Non-smokers | 1.00 | 0.43 (0.13-1.43) | 0.65 (0.24-1.79) | 0.82 (0.32-2.14) | 0.905 |
|  |  | Smokers | 1.00 | 2.26 (0.44-11.67) | 2.82 (0.57-13.97) | 2.03 (0.34-12.18) | 0.387 |
|  | Adjusted risk† | Non-smokers | 1.00 | 0.45 (0.13-1.51) | 0.56 (0.19-1.62) | 1.00 (0.37-2.72) | 0.894 |
|  |  | Smokers | 1.00 | 2.94 (0.53-16.30) | 5.08 (0.93-27.60) | 1.92 (0.27-13.58) | 0.374 |

†Adjusted for: age, height, BMI, ESR (log transformed), baseline glucose, cholesterol, physical activity, BP medication, social class, family history of diabetes, alcohol abuse *p <0.05 **p<0.01 ***p <0.001. P value for trend calculated using cox regression models (1 d.f)

**Table S7c: Hazard ratios of diabetes mellitus by quartiles of FEV_1_ %predicted in males: Stratified by follow up time and smoking status (10324 non-smokers and 9971 smokers)**

| **Follow up time (years)**  **(n= number of incident DM events)** |  |  | **Q4**  **(reference)** | **Q3** | **Q2** | **Q1** | **P value for trend** |
| --- | --- | --- | --- | --- | --- | --- | --- |
| **Non-smokers** |  |  | ≥109.50 | 99.09-109.49 | 88.66-99.09 | ≤88.66 |  |
| **Smokers** |  |  | ≥102.34 | 91.92-102.34 | 80.89-91.92 | ≤80.89 |  |
|  |  |  |  |  |  |  |  |
| 0-10 years  Non-smokers (n=169)  Smokers (n=196) | Unadjusted risk | Non-smokers | 1.00 | 1.22 (0.73-2.03) | 1.33 (0.81-2.19) | 2.73 (1.75-4.24)*** | <0.001 |
|  |  | Smokers | 1.00 | 0.70 (0.44-1.11) | 1.03 (0.67-1.56) | 1.89 (1.30-2.73)** | <0.001 |
|  | Adjusted risk† | Non-smokers | 1.00 | 1.18 (0.71-1.97) | 1.17 (0.71-1.93) | 1.89 (1.20-2.97)** | 0.003 |
|  |  | Smokers | 1.00 | 0.65 (0.41-1.03) | 0.90 (0.59-1.38) | 1.32 (0.90-1.92) | 0.035 |
|  |  |  |  |  |  |  |  |
| 10-20 years  Non-smokers (n=482)  Smokers (n=577) | Unadjusted risk | Non-smokers | 1.00 | 0.92 (0.70-1.21) | 1.03 (0.79-1.34) | 1.73 (1.35-2.20)*** | <0.001 |
|  |  | Smokers | 1.00 | 1.27 (0.98-1.65) | 1.54 (1.20-1.98)** | 2.19 (1.73-2.79)*** | <0.001 |
|  | Adjusted risk† | Non-smokers | 1.00 | 0.92 (0.69-1.21) | 0.94 (0.71-1.23) | 1.41 (1.10-1.80)** | 0.003 |
|  |  | Smokers | 1.00 | 1.24 (0.96-1.61) | 1.48 (1.15-1.91)** | 1.85 (1.45-2.35)*** | <0.001 |
|  |  |  |  |  |  |  |  |
| 20-30 years  Non-smokers (n=1001)  Smokers (n=983 ) | Unadjusted risk | Non-smokers | 1.00 | 1.19 (0.98-1.43) | 1.55 (1.30-1.86)*** | 1.49 (1.24-1.79)*** | <0.001 |
|  |  | Smokers | 1.00 | 1.16 (0.97-1.38) | 1.17 (0.98-1.41) | 1.52 (1.27-1.82)*** | <0.001 |
|  | Adjusted risk† | Non-smokers | 1.00 | 1.17 (0.97-1.42) | 1.45 (1.21-1.74)*** | 1.35 (1.12-1.63)** | <0.001 |
|  |  | Smokers | 1.00 | 1.13 (0.94-1.35) | 1.13 (0.94-1.35) | 1.42 (1.19-1.70)*** | 0.001 |
|  |  |  |  |  |  |  |  |
| >30 years  Non-smokers (n=180)  Smokers (n=165) | Unadjusted risk | Non-smokers | 1.00 | 0.96 (0.63-1.48) | 1.16 (0.77-1.76) | 1.34 (0.89-2.02) | 0.106 |
|  |  | Smokers | 1.00 | 1.17 (0.76-1.78) | 1.13 (0.73-1.75) | 1.50 (0.96-2.33) | 0.111 |
|  | Adjusted risk † | Non-smokers | 1.00 | 0.98 (0.64-1.52) | 1.12 (0.74-1.71) | 1.27 (0.84-1.91) | 0.209 |
|  |  | Smokers | 1.00 | 1.21 (0.79-1.85) | 1.15 (0.74-1.79) | 1.49 (0.96-2.32) | 0.117 |

†Adjusted for: age, height, BMI, ESR (log transformed), baseline glucose, cholesterol, physical activity, BP medication, social class, family history of diabetes, alcohol abuse *p <0.05 **p<0.01 ***p <0.001. P value for trend calculated using cox regression models (1 d.f)

**Table S7d Hazard ratios of diabetes mellitus by quartiles of FEV_1_ %predicted in females: Stratified by follow up time and smoking status (4065 non-smokers, 3351 smokers)**

| **Follow up time (years)**  **(n= number of incident DM events)** |  |  | **Q4**  **(reference)** | **Q3** | **Q2** | **Q1** | **P value for trend** |
| --- | --- | --- | --- | --- | --- | --- | --- |
| **Non-smokers** |  |  | ≥110.10 | 100.27-110.08 | 90.38-100.27 | ≤90.38 |  |
| **Smokers** |  |  | ≥101.32 | 90.63-101.31 | 79.18-90.62 | ≤79.18 |  |
|  |  |  |  |  |  |  |  |
| 0-10 years  Non-smokers (n=62)  Smokers (n=73) | Unadjusted risk | Non-smokers | 1.00 | 0.90 (0.38-2.13) | 1.73 (0.82-3.64) | 2.03 (0.98-4.18) | 0.016 |
|  |  | Smokers | 1.00 | 1.70 (0.83-3.47) | 1.53 (0.73-3.17) | 1.98 (0.98-3.97) | 0.089 |
|  | Adjusted risk† | Non-smokers | 1.00 | 0.86 (0.36-2.05) | 1.65 (0.77-3.51) | 1.30 (0.62-2.77) | 0.268 |
|  |  | Smokers | 1.00 | 1.64 (0.80-3.39) | 1.38 (0.66-2.90) | 1.23 (0.60-2.49) | 0.843 |
|  |  |  |  |  |  |  |  |
| 10-20 years  Non-smokers (n=204)  Smokers (n=191) | Unadjusted risk | Non-smokers | 1.00 | 1.29 (0.84-2.00) | 1.50 (0.98-2.29) | 1.99 (1.33-2.98)** | 0.001 |
|  |  | Smokers | 1.00 | 1.14 (0.74-1.75) | 1.27 (0.83-1.93) | 1.73 (1.15-2.58)** | 0.006 |
|  | Adjusted risk† | Non-smokers | 1.00 | 1.27 (0.82-1.97) | 1.52 (0.99-2.33) | 1.65 (1.09-2.49)* | 0.013 |
|  |  | Smokers | 1.00 | 1.08 (0.71-1.67) | 1.21 (0.79-1.84) | 1.24 (0.82-1.86) | 0.265 |
|  |  |  |  |  |  |  |  |
| 20-30 years  Non-smokers (n=227)  Smokers (n=192) | Unadjusted risk | Non-smokers | 1.00 | 1.02 (0.68-1.53) | 1.38 (0.94-2.02) | 1.73 (1.19-2.51)** | 0.001 |
|  |  | Smokers | 1.00 | 1.09 (0.74-1.61) | 1.02 (0.69-1.53) | 1.35 (0.90-2.02) | 0.222 |
|  | Adjusted risk† | Non-smokers | 1.00 | 0.94 (0.63-1.42) | 1.25 (0.85-1.85) | 1.47 (1.01-2.15)* | 0.015 |
|  |  | Smokers | 1.00 | 1.04 (0.71-1.54) | 0.95 (0.63-1.43) | 0.98 (0.65-1.49) | 0.827 |
|  |  |  |  |  |  |  |  |
| >30 years  Non-smokers (n=28)  Smokers (n=16) | Unadjusted risk | Non-smokers | 1.00 | 1.57 (0.48-5.09) | 1.04 (0.30-3.55) | 1.18 (0.36-3.94) | 0.919 |
|  |  | Smokers | 1.00 | 0.29 (0.03-2.78) | 2.21 (0.59-8.35) | 1.93 (0.43-8.65) | 0.110 |
|  | Adjusted risk† | Non-smokers | 1.00 | 1.56 (0.47-5.12) | 1.01 (0.29-3.60) | 1.27 (0.37-4.34) | 0.956 |
|  |  | Smokers | 1.00 | 0.28 (0.03-2.87) | 2.04 (0.52-8.04) | 2.26 (0.48-10.66) | 0.096 |

†Adjusted for: age, height, BMI, ESR (log transformed), baseline glucose, cholesterol, physical activity, BP medication, social class, family history of diabetes, and alcohol abuse. *p <0.05 **p<0.01 ***p <0.001. P value for trend calculated using cox regression models (1 d.f)

**Table S8: Hazard ratios of diabetes mellitus by FEV_1_/FVC ratio: Stratified by sex (Males= 20 295, females, 7416)**

| **Diabetes events for males and females** |  | **FEV_1_/FVC ≥70%** | **FEV_1_/FVC <70%** | **P value** |
| --- | --- | --- | --- | --- |
|  |  |  |  |  |
| Males (n=3753) | Unadjusted risk | 1.00 | 1.15 (1.05-1.25) | 0.002 |
|  | Adjusted risk* | 1.00 | 1.06 (0.98-1.16) | 0.162 |
|  |  |  |  |  |
| Females (n=993) | Unadjusted risk | 1.00 | 1.11 (0.90-1.36) | 0.343 |
|  | Adjusted risk* | 1.00 | 0.99 (0.81-1.22) | 0.952 |

*Adjusted for: age, height, BMI, smoking status, ESR (log transformed), baseline glucose, cholesterol, physical activity, BP medication, social class, family history of diabetes, and alcohol abuse.
